# Long-Term Follow-Up of Patients Immunized with AN1792: Reduced Functional Decline in Antibody Responders

**DOI:** 10.2174/156720509787602852

**Published:** 2009-04

**Authors:** Bruno Vellas, R Black, Leon J Thal, Nick C Fox, M Daniels, G McLennan, C Tompkins, C Leibman, M Pomfret, Michael Grundman, for the AN1792 (QS-21)-251 Study Team

**Affiliations:** Department of Geriatrics INSERM U 558, Toulouse University Hospital Center, Toulouse, France (BV); Wyeth Research, Philadelphia, Pennsylvania (RB, MP); University of California, San Diego, California (LT-deceased); Institute of Neurology, University College London, UK (NF); Elan Pharmaceuticals, South San Francisco, California (MD, MG, GMcL, CT, CL), USA

**Keywords:** Alzheimer’s disease, Immunotherapy, Aβ, amyloid, NTB.

## Abstract

**Background:**

Immunization of patients with Alzheimer’s disease (AD) with synthetic amyloid-β peptide (Aβ_42_) (AN1792) was previously studied in a randomized, double-blind, placebo-controlled phase 2a clinical trial, Study AN1792(QS-21)-201. Treatment was discontinued following reports of encephalitis. One year follow-up revealed that AN1792 antibody responders showed improvements in cognitive measures as assessed by the neuropsychological test battery (NTB) and a decrease in brain volume compared with placebo.

**Methods:**

A follow-up study, Study AN1792(QS-21)-251, was conducted to assess the long-term functional, psychometric, neuroimaging, and safety outcomes of patients from the phase 2a study 4.6 years after immunization with AN1792. The results were analyzed by comparing patients originally identified as antibody responders in the AN1792 phase 2a study with placebo-treated patients.

**Results:**

One hundred and fifty-nine patients/caregivers (30 placebo; 129 AN1792) participated in this follow-up study. Of the 129 AN1792-treated patients, 25 were classified in the phase 2a study as antibody responders (anti-AN1792 titers ≥1:2,200 at any time after the first injection). Low but detectable, sustained anti-AN1792 titers were found in 17 of 19 samples obtained from patients classified as antibody responders in the phase 2a study. No detectable anti-AN1792 antibodies were found in patients not classified as antibody responders in the phase 2a study. Significantly less decline was observed on the Disability Assessment for Dementia scale among antibody responders than placebo-treated patients (*p*=0.015) after 4.6 years. Significant differences in favor of responders were also observed on the Dependence Scale (*p*=0.033). Of the small number of patients who underwent a follow-up MRI, antibody responders showed similar brain volume loss during the follow-up period subsequent to the AN1792 phase 2a study compared with placebo-treated patients.

**Conclusions:**

Approximately 4.6 years after immunization with AN1792, patients defined as responders in the phase 2a study maintained low but detectable, sustained anti-AN1792 antibody titers and demonstrated significantly reduced functional decline compared with placebo-treated patients. Brain volume loss in antibody responders was not significantly different from placebo-treated patients approximately 3.6 years from the end of the original study. No further cases of encephalitis were noted. These data support the hypothesis that Aβ immunotherapy may have long-term functional benefits.

## INTRODUCTION

Alzheimer’s disease (AD) is a neurodegenerative disorder that represents the leading cause of dementia in the elderly, with approximately 27 million AD patients worldwide. The global prevalence of AD is expected to quadruple to approximately 106 million patients by 2050 [1]. Treatment options to delay or halt the progression of AD to dementia are highly desirable. Immunotherapy with human amyloid-β (Aβ) 1-42 peptide (AN1792) stimulated the clearance of amyloid plaques and prevented AD-associated cognitive decline in a mouse model of AD [2]. Efficacy observed after immunization with AN1792 in the mouse model led to the strategy of targeted Aβ immunotherapy for removal and clearance of Aβ from the brains of AD patients.

Preclinical studies in several species demonstrated the safety, tolerability, and activity of AN1792 in combination with the adjuvant QS-21 [2-6]. Phase 1 studies demonstrated that the optimal dose combination for eliciting an anti-AN1792 antibody response was AN1792 225 µg and QS-21 50 µg [7]. Accordingly, a double-blind, placebo-controlled, multi-center phase 2a study (Study 201) was initiated to evaluate the safety, tolerability, and pilot efficacy of AN1792 in patients with mild-to-moderate AD. Study drug administration was discontinued in January 2002 after the first reports of meningoencephalitis [8]. The protocol was amended to monitor all patients for 12 months from the first dose of drug, while maintaining the study blind to determine the safety and tolerability of AN1792. Efficacy measures continued to be assessed.

At the end of the 1-year follow-up assessments in the phase 2a study, AN1792-treated patients who were antibody responders (anti-Aβ titers ≥1:2,200) showed improvements in cognitive measures as assessed by a 9-component neuropsychological test battery (NTB) z-score, a composite of tests assessing memory and executive function. Furthermore, antibody responders showed a reduction in cerebrospinal fluid (CSF) tau levels compared with placebo-treated patients, which suggested a reduced level of neurodegeneration compared with baseline [9]. Volumetric brain MRI results revealed a decrease in whole brain volume (WBV) and an increase in ventricular volume in antibody responders compared with placebo-treated patients. Interestingly, despite the greater loss of brain volume, antibody responders maintained a cognitive advantage compared with placebo-treated patients, implying that the extra brain volume reduction seen in the responders was not due to neuronal loss [10]. Autopsy of 4 patients who were treated with AN1792 (2 with encephalitis and 2 without encephalitis) showed evidence of amyloid plaque clearance [11-13].

This follow-up study (Study 251) was designed to evaluate whether those patients who were antibody responders in the phase 2a study had altered clinical outcomes or rates of brain atrophy after long-term follow up in the absence of treatment compared with placebo-treated patients. Evidence of clinical benefit would provide further support for Aβ immunotherapy as a potential treatment approach in AD. 

## METHODS

### Patients

Patients were required to have participated in the phase 2a study and to have provided appropriate written informed consent to participate in the follow-up analysis [9]. Patients eligible for entry to the phase 2a study were 50 to 85 years of age, met the criteria for a diagnosis of probable AD as defined by the National Institute of Neurologic and Communicative Disorders and Stroke–AD and Related Disorders Association, and had an MRI brain scan supporting the clinical diagnosis of AD. Additional inclusion criteria were a score of 15 to 26 on the Mini-Mental State Examination (MMSE), a Rosen-Modified Hachinski Ischemic score of ≤4, and written informed consent from the patient and the patient’s caregiver for the original protocol and subsequent amendments. Patients were excluded if they had clinically significant neurologic disease (other than AD) that might affect cognition, a major psychiatric disorder, systemic illness or symptoms that could affect the patient’s ability to complete the study, a Hamilton Psychiatric Rating Scale for Depression (17 item) score of >12, used anticonvulsant, antiparkinsonian, anticoagulant, narcotic, or immunosuppressive medication within 3 months prior to baseline, used medication with the potential to affect cognition (unless maintained on a stable low-to-moderate–dose regimen for at least 3 months prior to baseline), or used medication for cognitive enhancement other than a stable dosing regimen of an acetylcholinesterase inhibitor (≥6 months).

### Study 201 Design and Treatment

This randomized, placebo-controlled, double-blind, phase 2a clinical trial, reported by Gilman and colleagues in 2005, was conducted at 28 centers in the United States and Europe between September 2001 and December 2002 [9]. A total of 372 patients with mild-to-moderate AD were randomly assigned in a double-blind manner to receive treatment with a suspension of AN1792 225 µg (Elan Pharmaceuticals, Inc., South San Francisco, CA) and QS-21 50 µg (Antigenics, Framingham, MA) containing 0.4% polysorbate-80, or normal saline (placebo) in a 4:1 ratio. A 6-member independent Data Safety Monitoring Committee assessed the safety of the study drug throughout Study 201.

In each group, treatment was administered as a single 0.5 mL intramuscular (IM) injection and dosing was to occur on Day 0 and at Months 1, 3, 6, 9, and 12 according to the original protocol. Due to the early discontinuation of the immunization, patients received only 1 to 3 injections [9]. All patients enrolled in the study (including those who discontinued early) were invited to participate in the safety follow-up period, which continued blinded and lasted until Month 12 (ie, at least 9 months after their last dose of study treatment). 

### Follow-Up Study 251 Design

This was a multi-center out-patient follow-up study in patients with AD who had participated in the phase 2a study described above [9]. The follow-up study was conducted at 23 centers in the United States and Europe between January and October 2006. No study medication was dispensed in this follow-up study.

An initial telephone contact was made to assess interest in participation. Subsequently, a clinic visit was conducted after obtaining informed consent. At this visit, a blood sample to assess serum anti-AN1792 antibody titers and plasma Aβ concentration was collected. The following functional and cognitive assessments were performed: Disability Assessment for Dementia (DAD), Dependence Scale, Clinical Dementia Rating–Sum of Boxes (CDR-SOB), MMSE, NTB, and Alzheimer’s Disease Assessment Scale Cognitive Subscale (ADAS-Cog). A volumetric brain MRI scan was also performed. Changes in the patients’ living situation since the beginning of the phase 2a study were queried. Any serious adverse events (SAEs) that occurred after the last visit in the initial phase 2a study were assessed, and the relationship to study drug was determined by the investigator. 

A home visit or telephone contact, in lieu of the clinic visit, was conducted if the patient could not attend the clinic. In these patients, the DAD, Dependence Scale, CDR-SOB, and questions regarding changes in living situation were administered over the telephone. The telephone contact was also used to collect patient information regarding any SAEs that may have occurred since the previous phase 2a study. Antibody titers were not assayed and follow-up MRIs were not conducted in this group that could not attend clinic. 

The clinical raters performing the cognitive and functional instruments in the AN1792-251 follow up study performed these tests without knowledge of the patient’s group assignment or antibody status. Patients and caregivers were given access to treatment assignment upon request at the end of the AN1792-201 phase 2a study, however, they were not provided information regarding whether they were antibody responders or nonresponders. 

Site-specific, local, independent ethics committees approved the protocol, amendments, and related informed consent forms prior to implementation of each study. The studies were conducted in accordance with the International Conference on Harmonization Tripartite Guidelines on Good Clinical Practice and in compliance with the Declaration of Helsinki 1964 as modified in October 2000. 

### Study 251 Outcome Measures and Statistical Analyses

An enzyme-linked immunosorbent assay (ELISA) was used to determine total anti-AN1792 immunoglobulin (Ig)G levels in follow-up study baseline serum samples. The ELISA had a lower limit of detection of 1:50 for serum IgG. Patients who achieved a serum anti-AN1792 titer ≥1:2,200 any time after the first injection in the phase 2a study were considered antibody responders. A titer ≥1:2,200 was preselected as the minimum threshold anti-AN1792 titer likely to be of significant clinical benefit, based on preclinical data (Elan Pharmaceuticals, Inc., data on file).

### Functional, Dependence, and Global Endpoints

The DAD scale was administered to caregivers to measure the patients’ performance of instrumental and basic activities of daily living over a 2-week period before the scheduled follow-up visit [14]. For each activity, questions were asked to evaluate the patients’ performance on initiation, planning and organization, and effectiveness. Change from phase 2a study baseline for the DAD score was analyzed using an analysis of covariance (ANCOVA) model with change from phase 2a study baseline DAD score as the response, study group (Study 201 antibody responder classification vs. placebo) as a factor, and phase 2a study baseline DAD score as a covariate.

The Dependence Scale [15], also completed by the caregiver, asks 13 questions assessing the required amount of assistance or care from others needed by the patient. The scale is scored as a sum of items, with patients having the least degree of dependence scored a 0 and patients with the highest level of need receiving a 15. Comparisons of the total Dependence Scale scores in the follow-up study were performed using analysis of variance (ANOVA).

The CDR-SOB scale measures patient impairment due to cognitive loss by evaluating 6 categories or box scores: memory, orientation, judgment and problem solving, community affairs, homes and hobbies, and personal care [16]. The rating is based on interviews conducted with both the patient and the caregiver. Change from baseline for the CDR-SOB score was analyzed using an ANCOVA model with change from phase 2a study baseline CDR-SOB score as the response, study group as a factor, and phase 2a study baseline CDR-SOB score as a covariate.

### Cognitive Endpoints

The NTB was used to assess cognitive and executive function, and consisted of 6 memory tests (Wechsler Memory Visual Paired Associates–Immediate and Delayed Recall; Wechsler Memory Verbal Paired Associates–Immediate and Delayed Recall; Rey Auditory Verbal Learning Test–Immediate and Delayed Recall) and 3 executive function tests (Wechsler Memory–Digit Span; Controlled Word Association Test [FAS]; Category Fluency Test [animals]) [17]. Raw scores on each of the 9 NTB tests were converted to z-scores using the baseline means and standard deviations (SDs) of phase 2a study participants for each test. The resultant z-scores were averaged to obtain a composite z-score, incorporating all 9 NTB tests. The NTB was further analyzed in subgroups of the overall composite NTB z-score, including the all memory z-score (utilizing all 6 memory tests) and executive function z-score (utilizing the 3 executive function tests). Change from baseline was calculated as the post-baseline composite z-score minus the phase 2a study baseline score, such that a positive change indicates an improvement from baseline. NTB scores were analyzed using an ANCOVA model with change from phase 2a study baseline NTB score as the response, study group as a factor, and phase 2a study baseline NTB score as a covariate. 

In addition to the NTB, other cognitive endpoints included the MMSE and the ADAS–Cog [18, 19]. Analysis of the MMSE and ADAS-Cog were handled similarly to the NTB, using an ANCOVA model with change from the phase 2a study baseline score as the response, study group as a factor, and phase 2a study baseline score as a covariate. 

### Additional Endpoints

Caregivers were queried as to where the patients lived at the start and finish of the phase 2a study and at the time of the follow-up study. The frequency and percentage of patients who remained in their own home or who entered a long-term care facility or nursing home from the beginning of the phase 2a study were determined. 

Changes in brain, ventricular, and hippocampal volume were determined by MRI methods previously described [10]. Brain boundary shift integral was only calculated on scan pairs that did not have a significant acquisition change between time points. All decisions on patient suitability for scan acquisition or inclusion for analysis were made blind to treatment status. Similarly, all MRI measurements were performed blind to treatment status. Change on MRI was evaluated from baseline as well as from the final MRI visit in the phase 2a study (Month 12). In each case, as appropriate, the baseline volume or final MRI volume was included in the model as a covariate. 

### Safety

Information about SAEs that occurred since the end of the phase 2a study was collected and summarized. Patients’ vital status (ie, alive or dead) was also recorded and compared between treatment groups.

## RESULTS

### Patients

**Fig. (1):**
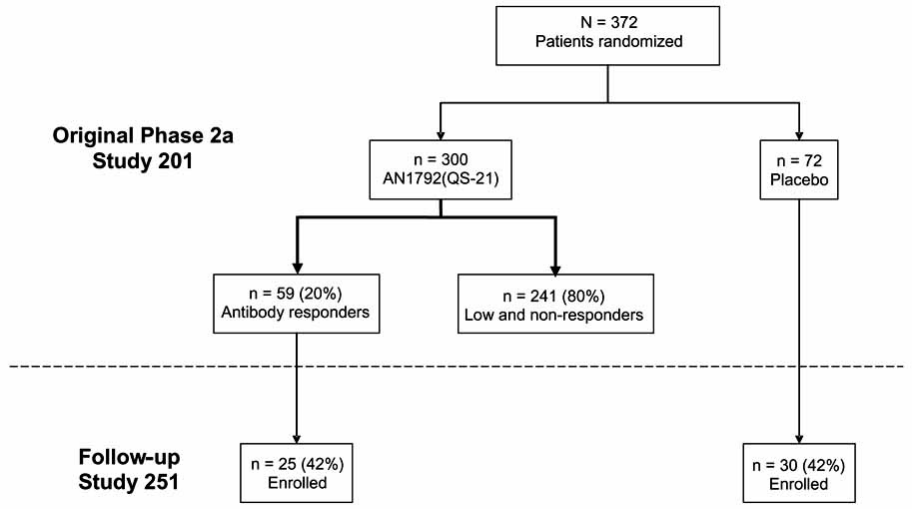
Disposition of patients classified as antibody responders and placebo-treated patients enrolled in Study 251. Patients were considered antibody responders in Study 201 if they had a serum anti-AN1792 IgG (total) titer ≥1:2,200 at any time after injection 1.

Of 372 patients treated in the phase 2a study (300 active; 72 placebo), a total of 264 (71.0%) patients/caregivers were contacted to determine interest in follow-up study participation. One hundred and fifty-nine patients/caregivers agreed to participate in the follow-up study (30 placebo; 129 treated with AN1792 + QS-21). Of the original 59 subjects who were classified in the phase 2a study as antibody responders (anti-AN1792 titers ≥1:2,200 at any time after the first injection), 25 (42.4%) agreed to participate in the follow-up study compared with 30/72 (41.7%) of the placebo-treated patients (Fig. **1**). In addition, 104/241 (43.2%) of patients classified as low or non-responders from Study 201 were enrolled in this follow-up study; however, data gathered from these patients and caregivers are beyond the objectives and scope of the present communication. Patient demographics and baseline characteristics were similar between antibody responders and placebo-treated patients at the start of this follow-up study (Table **1**). 

A substantial portion of patients and/or caregivers were not contacted for participation in this follow-up study. A number of patients not contacted were known to be dead or were lost to follow up. Similar numbers of patients/ caregivers were not able to be contacted in both the antibody responder group (n=19 [32.2%]) and the placebo group (n=22 [30.6%]) (Table **1**). Of the live patients and their caregivers who were contacted to participate in this follow-up study, fewer antibody responders refused to participate (8/39, or 20.5%), compared with 15/48 (31.3%) of placebo-treated patients (Table **1**).

The majority of patients enrolled in this follow-up study were taking concomitant acetylcholinesterase inhibitors or memantine. The proportion of patients taking concomitant medications was similar between antibody responders and placebo-treated patients. Twenty-two of 30 (73.3%) placebo-treated patients were taking concomitant acetylcholinesterase inhibitors compared with 19/25 (76.0%) of antibody responders. Fifteen of 30 (50.0%) placebo-treated patients were taking concomitant memantine, compared with 15/25 (60.0%) of antibody responders.

Mean time of follow up, either from baseline or from the final study visit in the original Phase 2a study to final assessment in this analysis, was similar across treatment and response groups (Table **1**). 

### Immunologic Findings

Of the 300 patients randomized to receive AN1792 in the phase 2a study, 59 (19.7%) were classified as antibody responders (having total serum anti-AN1792 IgG titers ≥1:2,200 at any time after the first injection)—25 of these antibody responders agreed to participate in this follow-up study. Of these participating antibody responders, 19 submitted a blood sample for antibody testing. Seventeen of the 19 tested antibody responders (89.5%) had a low but persistent residual mean anti-AN1792 antibody titer, with a geometric mean of 1:331.5 compared with non-detectable levels in all 19 placebo-treated patients who submitted a blood sample (*p*<0.001). No detectable anti-AN1792 antibodies were found in patients not classified as antibody responders in the phase 2a study. Antibody responders retained low but persistent levels of anti-AN1792 antibodies after approximately 4.6 years despite receiving only 1 to 3 of the planned injections in the original Phase 2a study.

### Changes in Functional, Dependence, and Global Measures

#### DAD

Caregivers provided DAD ratings for 27/30 placebo-treated patients (90.0%) and 24/25 antibody responders (96.0%). After approximately 4.6 years of follow up, antibody responders demonstrated a 25.0% lower decline in activities of daily living as determined by the DAD compared with placebo-treated patients (mean change ± SD of -56.34 ± 28.45 for placebo vs -42.28 ± 30.88 for antibody responders, *p*=0.015). 

#### Dependence Scale

Caregivers provided Dependence Scale ratings for 28/30 placebo-treated patients (93.3%) and 24/25 antibody responders (96.0%). Antibody responders demonstrated a 17.6% lower mean score in caregiver dependence compared with placebo-treated patients (mean score ± SD of 10.2 ± 2.6 for placebo vs 8.4 ± 3.4 for antibody responders, *p*=0.033). 

#### CDR-SOB

CDR-SOB ratings were obtained for 24/30 placebo-treated patients (80.0%) and 22/25 antibody responders (88.0%). Similar to the DAD and Dependence Scale, antibody responders from the phase 2a study showed 20.2% less decline on the CDR-SOB scale compared with placebo, although this difference was not statistically significant (mean change ± SD of 7.63 ± 4.48 for placebo vs 6.09 ± 4.75 for antibody responders, *p*=0.185). 

### Cognitive Function

#### NTB

The NTB as well as the other cognitive instruments were only administered in follow up to those patients who were available for testing and capable of being tested on these instruments. At the time of the follow-up study, the NTB was attainable in only 10/30 placebo-treated patients (33.3%) and 13/25 antibody responders (52.0%). No significant differences were observed in the change from baseline for the overall NTB 9-component z-score between antibody responders and placebo-treated patients (mean change ± SD of -0.751 ± 0.560 for placebo vs -0.597 ± 0.626 for antibody responders, *p*=0.440). Of the NTB z-score components, the Rey Auditory Verbal Learning Test – Delayed Recall component showed reduced decline among antibody responders (mean change ± SD of -1.096 ± 1.165 for placebo vs 0.114 ± 1.138 for antibody responders, *p*=0.046).

#### MMSE and ADAS-Cog

An MMSE was attainable in 18/30 placebo-treated patients (60.0%) and 20/25 antibody responders (80.0%). No significant differences were observed between antibody responders compared with placebo-treated patients after 4.6 years of follow up (mean change ± SD of -8.3 ± 5.8 for placebo vs -8.0 ± 7.8 for antibody responders, *p*=0.719). 

The ADAS-Cog was attainable in 11/30 placebo-treated patients (36.7%) and 16/25 antibody responders (64.0%). No significant differences in ADAS-Cog score were observed between placebo-treated patients and antibody responders after approximately 4.6 years of follow up (mean change ± SD of 14.4 ± 14.2 for placebo vs 14.3 ± 14.0 for antibody responders, *p*=0.616). 

#### Changes in Living Situation

Changes in living situation were available for all participating subjects in the follow-up study. After approximately 4.6 years, 19/25 (76.0%) antibody responders remained in their own home compared with 16/30 (53.3%) placebo-treated patients (*p*=0.099). Similarly, only 4/25 (16.0%) antibody responders were in long-term care institutions after approximately 4.6 years compared with 9/30 (30.0%) placebo-treated patients (*p*=0.341).

### MRI Brain Volume Analysis

Patients who were antibody responders in the phase 2a study demonstrated increased WBV loss compared with placebo-treated patients, with no associated increase in cognitive decline [10]. This follow-up study compared changes in brain volume measurements between antibody responders and placebo-treated patients from the last visit in the phase 2a study with those observed at the time of the follow-up study visit. MRIs were available for 8/30 (26.7%) placebo-treated patients and 8/25 (32.0%) antibody responders. From the time of the last MRI in the original phase 2a study, antibody responders demonstrated no significant differences in WBV loss compared with placebo-treated patients (mean percent change ± SD of -5.11% ± 3.96% for placebo vs -5.66% ± 2.62% for antibody responders, *p*=0.854). No significant difference on brain boundary shift integral (mean percent change ± SD of 4.69% ± 2.36% for placebo vs 6.61% ± 2.86% for antibody responders, *p*=0.211) was observed; however, because of changes in scan acquisition, the boundary shift integral was only available for 7 placebo-treated patients and 6 antibody responders. There was also no significant difference in the percentage increase in ventricular volume, expressed as a percentage of baseline whole-brain volume, between placebo-treated patients and antibody responders (mean percent change ± SD of 1.25% ± 0.70% for placebo vs 2.00% ± 0.88% for antibody responders, *p*=0.285) after 3.6 years from the last MRI in the phase 2a study. Similarly, there was no significant difference in either left hippocampal loss (mean percent change ± SD of -12.44% ± 8.78% for placebo vs -15.28% ± 7.62% for antibody responders, *p*=0.436) or right hippocampal loss (mean percent change ± SD of -10.57% ± 7.58% for placebo vs -13.51% ± 5.74% for antibody responders, *p*=0.402).

This follow-up study also compared changes in brain volume measurements between antibody responders and placebo-treated patients from the baseline MRI in the phase 2a study with those observed at the time of the follow-up study. MRI scan pairs were available for 7/30 (23.3%) placebo-treated patients and 8/25 (32.0%) antibody responders at baseline. Antibody responders demonstrated no significant differences in WBV loss compared with placebo-treated patients after approximately 4.6 years from the first MRI performed in the original phase 2a study (mean percent change ± SD of -6.24% ± 4.58% for placebo vs -7.94% ± 1.70% for antibody responders, *p*=0.383). Brain boundary shift integral measures were available for 6 placebo-treated patients and 5 antibody responders, and demonstrated no significant difference (mean percent change ± SD of 5.10% ± 2.70% for placebo vs 8.48% ± 3.81% for antibody responders, *p*=0.119). Similarly, there was no significant difference in either left hippocampal loss (mean percent change ± SD of -13.54% ± 8.47% for placebo vs -17.03% ± 8.76% for antibody responders, *p*=0.346) or right hippocampal loss (mean percent change ± SD of -10.86% ± 6.49% for placebo vs -14.74% ± 5.92% for antibody responders, *p*=0.164). However, a significant difference in ventricular volume enlargement, expressed as a percentage of baseline whole-brain volume, was observed from baseline after approximately 4.6 years in this subgroup of patients with MRI follow up (mean percent change ± SD of 1.39% ± 0.82% for placebo vs 2.83% ± 1.29% for antibody responders, *p*=0.021).

### Safety

Patients and caregivers who enrolled in the follow-up study were asked to provide information on any SAEs that occurred from the time of their final visit in the phase 2a study. There was a similar overall incidence of SAEs for placebo-treated patients (33.3%) compared with antibody responders (40.0%). No SAEs were considered by the investigator to be related to AN1792. No cases of encephalitis were reported in this follow-up study. No deaths were considered related to study drug by the investigator. Vital status information was available for 53/72 (73.6%) of placebo-treated patients and 45/59 (76.3%) antibody responders who were participants in the original phase 2a study. Survival was not significantly different between antibody responders and placebo-treated patients. For subjects in whom vital status information was available, 44/53 (83.0%) placebo-treated patients remained alive compared with 32/45 (71.1%) of antibody responders (*p*=0.220).

## DISCUSSION

The comparisons conducted in this AN1792 follow-up study were performed between patients classified as antibody responders and those treated with placebo in the original phase 2a study. The prospectively defined goal of these analyses was to determine if benefits might accrue over time in patients who developed prospectively defined antibody titers (≥1:2,200) that were considered adequately therapeutic in the phase 2a study.

This follow-up study suggests that 1 to 3 doses of AN1792 administered to patients with mild-to-moderate AD may have had long-term clinical benefits in patients who developed anti-AN1792 antibodies. Patients classified as antibody responders at the end of the phase 2a study retained low but persistent anti-AN1792 antibody titers after approximately 4.6 years. Compared with placebo-treated patients, antibody responders demonstrated significantly less impairment in activities of daily living and significantly less dependence on caregivers. A higher percentage of antibody responders remained at home and a lower percentage were institutionalized. These results provide further support for the Aβ immunotherapeutic approach in patients with mild-to-moderate AD.

In contrast to testing of functional and dependence measures (for which caregiver assessments were available for the majority of participating subjects), fewer assessments of cognitive outcomes (including the NTB, MMSE, and ADAS-Cog) were able to be performed, as patients became less amenable to or were unable to undergo cognitive evaluation due to disease progression. For all cognitive outcomes, there were relatively fewer placebo-treated patients eligible for cognitive testing than antibody responders. Therefore, comparisons of cognitive function in this follow-up study may not be representative of the entire placebo-treated group, and may favor the assessment of placebo-treated patients who were less impaired and capable of undergoing cognitive testing. Despite this potential bias to include better-performing placebo-treated patients in the cognitive analyses, antibody responders tended to perform better on the memory component of the NTB. They demonstrated significantly less decline on a memory subtest of the NTB (Rey Auditory Verbal Learning Test – Delayed Recall) and similar declines compared with placebo on the other cognitive outcomes.

A similar overall incidence of SAEs for placebo-treated patients compared with antibody responders was observed. No SAEs were considered by the investigator to be treatment related. Although dosing in the phase 2a study was halted after 18 patients (6.0%) developed encephalitis, no new cases of encephalitis were reported after 4.6 years in this follow-up study, and similar survival was observed between treatment groups.

Antibody responders in the phase 2a study demonstrated increased brain volume loss and increased ventricular enlargement over 11 months of follow up. In the small sample of patients who had MRI scans in this follow-up study, brain volume loss in antibody responders from the time of the last phase 2a study visit was similar to changes observed in placebo-treated patients. Ventricular enlargement was still significantly greater in antibody responders when compared with baseline measurements in the phase 2a study, but not when compared with the final scan. When compared with the final scan performed in the original study we were not able to show significant differences between antibody responders and placebo-treated patients on any MRI measures (whole brain or hippocampal loss and ventricular enlargement). It is tempting to speculate that rates of loss were similar between the two groups after the end of the phase 2a study; however, due to the small numbers of subjects, the study had very limited power to detect any such differences. Although firm conclusions cannot be drawn, this suggests that there continues to be dissociation, as observed in our original report, of better cognitive or functional performance despite greater brain volume loss [10]. This implies that whatever accounted for the increased loss on MRI measures it was not neuronal degeneration.

Results from a phase 1 study of patients with mild-to-moderate AD treated with AN1792 demonstrated reduction in functional decline as measured by DAD that did not reach statistical significance until approximately 20 months [7]. The phase 2a study represents an independent patient group treated with AN1792. Differences in DAD score between antibody responders and placebo-treated patients similarly did not reach significance at 1 year, but did eventually show statistically significant differences after 4.6 years [9]. This finding is particularly noteworthy because only residual low titers were observed after long-term follow up. One obvious question this follow-up study raises is what clinical effects might have been observed had antibody responders retained a higher antibody titer (ie, ≥2,200) over the length of follow up.

The NTB scale, comprised of 9 well-known and validated cognitive tests, was utilized in the previous AN1792 phase 2a study [9, 17]. In the follow-up study reported here, a greater percentage of antibody responders performed the NTB than placebo-treated patients. Similarly, relatively fewer placebo-treated patients were administered the ADAS-Cog and MMSE compared with antibody responders. No apparent benefits were detected in antibody responders compared with placebo-treated patients among the testable subjects on the ADAS-Cog or MMSE. It is possible that amyloid-targeted immunotherapies have a greater effect on the retention of memory-related functions than on other cognitive domains. Alternatively, the lack of benefit seen on the MMSE and ADAS-Cog may be confounded if only the less impaired placebo-treated patients capable of undergoing cognitive testing were assessed. The latter explanation is supported by the significant treatment effects seen on the DAD and Dependence Scale in whom data on more patients was available.

A limitation of this study was that only a small proportion of the subjects could be reassessed this long after the start of the original phase 2a study; the proportion that could be restudied with MRI was inevitably even smaller. Nonetheless, it is interesting to note that after approximately 4.6 years, patients classified as responders to initial therapy with AN1792 retained detectable levels of anti-AN1792 antibody. Moreover, antibody responders demonstrated functional and dependence benefits compared with placebo-treated patients, with comparable safety and survival observed between treatment groups. No SAEs were considered treatment related, and no additional cases of encephalitis were reported. Placebo-treated patients and antibody responders did not demonstrate significant differences in loss of brain volume approximately 3.6 years from the end of the phase 2a study. These studies suggest that Aβ remains a valid target for the treatment of mild-to-moderate AD, demonstrating that strategies to reduce Aβ may have the potential to sustain important clinical benefits in patients with AD. A number of different immunotherapeutic approaches to the treatment of AD are currently being studied. 

## DISCLOSURES

C. Leibman and M. Grundman are employees of Elan Pharmaceuticals and have stock in the company. R. Black and M. Pomfret are employees of Wyeth Research. R. Black holds stock in the company. N. Fox has received honoraria from Elan Pharmaceuticals and Wyeth Research. The Dementia Research Centre has conducted MRI analyses sponsored by Elan Pharmaceuticals and Wyeth Research.

## Figures and Tables

**Table 1.** Patient Disposition and Demographics by Treatment and Response Group

	Antibody Responder	Placebo-treated
Patients enrolled in original Phase 2a Study 201 (n)	59	72
Who died in Study 201(n [%])	1 (1.7)	2 (2.8)
Patients or caregivers not contacted for participation in Study 251 (n [%])	19 (32.2)	22 (30.6)
Known dead (n [%])	5 (8.5)	3 (4.2)
Status unknown (n [%])	14 (23.7)	19 (26.4)
Patients or caregivers contacted for participation in Study 251 (n [%])	39 (66.1)	48 (66.7)
Dead, caregiver did not agree to participate (n [%])	6 (10.2)	3 (4.2)
Alive, did not agree to participate (n [%])	8 (13.6)	15 (20.8)
Dead, caregiver agreed to participate (n [%])	1 (1.7)	1 (1.4)
Alive, agreed to participate (n [%])	24 (40.7)	29 (40.3)
Total participation in Study 251, alive or dead (n [%])	25 (42.4)	30 (41.7)
Mean time to follow up in Study 251 from baseline in original Phase 2a Study 201 ± SD (years)	4.63 ± 0.12	4.61 ± 0.10
Mean time to follow up in Study 251 from end of original Phase 2a Study 201 ± SD (years)	3.66 ± 0.14	3.63 ± 0.14
Mean age ± SD (years), start of Study 251	74.9 ± 7.32	73.7 ± 8.65
Phase 2a Study 201 baseline mean MMSE ± SD, (n)	21.0 ± 3.3 (24)	19.8 ± 2.8 (29)
ApoE4 carriers, n (%)	20 (80.0)	17 (56.7)

Note: The denominators for patient disposition percentages are the numbers of patients enrolled in the original Phase 2a Study 201. The denominators for ApoE4 carrier percentages are the total numbers participating in Study 251, alive or dead.

## APPENDIX

The AN1792 (QS-21)-251 Study Team comprises:

Mark Brody, Brain Matters Research, Inc., Delray Beach, FL.

Jody Corey-Bloom, Alzheimer’s Disease Research Center, UCSD, La Jolla, CA.

Mercè Boada Rovira, Fundació ACE, Institut Català de Neurociències Aplicades, Barcelona, Spain.

Jean-Francois Dartigues, Hôpital Pellegrin–Tripode, Bordeaux, France.

Rachelle Doody, Baylor College of Medicine, Houston, TX.

Bruno Dubois, Hôpital La Pitié Salpêtrière, Paris, France.

Stephen Flitman, Xenoscience Inc., Phoenix, AZ.

Ana Frank-Garcia, Hospital Universitario La Paz, Madrid, Spain.

Daniel Grosz, Pharmacology Research Institute, Northridge, CA.

Christoph Hock, University of Zurich, Zurich, Switzerland.

Pierre Jouanny, Hôtel Dieu–CHU, Rennes, France.

Louis Kirby, Pivotal Research Centers, Peoria, AZ.

David Knopman, Mayo Clinic, Rochester, MN.

Bernard Laurent, Hôpital Bellevue, Saint-Etienne, France.

Bernard Michel, Hôpital Sainte-Marguerite, Marseille, France.

José Luis Molinuevo, Institut d’Investigació Biomèdica August Pi i Sunyer, Barcelona, Spain.

Florence Pasquier, Hôpital Roger Salengro, Lille, France.

Jordi Pena-Casanova, Hospital del Mar, Barcelona, Spain.

José Ribera Casado, Hospital Clinico, San Carlos, Madrid, Spain.

Ralph Richter, Clinical Pharmaceutical Trials, Inc., Tulsa, OK.

Anne-Sophie Rigaud, Broca Hospital, Paris, France.

Jacques Touchon, Hôpital Gui de Chaulliac, Montpellier, France.

Bruno Vellas, CHU La Grave–Casselardit, Toulouse, France.
